# Pediatric Hearing Thresholds Post-bacterial Meningitis

**DOI:** 10.3389/fsurg.2020.00036

**Published:** 2020-07-09

**Authors:** Mercy E. Jatto, Adebolajo A. Adeyemo, Segun A. Ogunkeyede, Ikeoluwa A. Lagunju, Onyekwere G. Nwaorgu

**Affiliations:** ^1^Department of Otorhinolaryngology, University College Hospital, Ibadan, Nigeria; ^2^Institute of Child Health, College of Medicine, University of Ibadan, Ibadan, Nigeria; ^3^Department of Otorhinolaryngology, College of Medicine, University of Ibadan, Ibadan, Nigeria; ^4^Department of Pediatrics, College of Medicine, University of Ibadan, Ibadan, Nigeria

**Keywords:** auditory brain stem response, bacterial meningitis, pediatric, hearing thresholds, otoacoustic emission

## Abstract

**Introduction:** Disabling hearing loss as a sequela of bacterial meningitis results from damage to the auditory system. This study was designed to ascertain the hearing thresholds in survivors of bacterial meningitis and the risk factors of hearing loss in childhood bacterial meningitis.

**Methodology:** One hundred and two children admitted and treated for bacterial meningitis were recruited prospectively along with 102 age- and sex-matched controls who had auditory evaluation using otoacoustic emission and auditory brain stem response tests 48 h prior to hospital discharge. This was also repeated at the follow-up clinic at 1 month after hospital discharge, irrespective of the initial hearing assessment results.

**Result:** There were 57 (55.9%) males and 45 (44.1%) females among the cases (mean age, 5.34 ± 4.40 years) and 55 (53.9%) males and 47 (46.1%) females among the controls (mean age, 5.31 ± 3.15 years). The prevalence of hearing loss was 30.4% among the cases, while it was 6.9% among the controls. The risk factors of hearing impairment in this study were the presence of anemia, leukocytosis, and hypoglycorrhachia.

**Conclusion:** Hearing impairment with varying degrees of severity is a frequent complication of bacterial meningitis in children.

## Introduction

Bacterial meningitis is the inflammation of the brain meninges (affecting the pia mater, arachnoid mater, and the subarachnoid space) due to bacterial infection (1). It is a prevalent global health problem despite the availability of vaccines against the usual pathogens responsible for the disease (2). An estimated 1.2 million cases of bacterial meningitis occur worldwide annually (3), with a high prevalence seen in low- and medium-income countries (LMICs), especially among African children (4, 5). The African continent accounts for ~50% of all bacterial meningitis cases (3). This is probably due to its young population, epidemics in the meningitis belt, and the high rates of endemicity of this disease (6). Children are more at risk of developing meningitis because of their immature immune system, malnutrition, poor immunization practices, and a high rate of poverty with its consequences (6, 7).

Africa is also the only region of the world that has cyclical epidemics of meningitis occurring commonly in the Africa meningitis belt, which extends from Senegal to Ethiopia, with North Eastern Nigeria inclusive. These epidemics usually occur during the dry season of the year, with major epidemics occurring once in every 5–12 years and with rates as high as 100–800 morbidities per 100,000 individuals (6). The three main causative organisms of bacterial meningitis after the neonatal period are *Haemophilus influenzae* type b, *Streptococcus pneumoniae*, and *Neisseria meningitides*. *S. pneumoniae* has the highest fatality and is more frequently associated with disabilities, followed by *H. influenzae* type b and *N. meningitides* (2, 8).

Meningitis has a significant mortality rate (3); however, neurocognitive sequelae with resultant disability are seen in 3–47% of cases among survivors (9–11). Hearing impairment is one of the leading neurological sequelae associated with bacterial meningitis (12), accounting for 60–90% of all cases of acquired post-lingual sensorineural hearing loss (SNHL) (13, 14). The release of by-products of inflammation, such as nitric oxide, superoxide, and peroxynitrite, contributes to the disruption of the blood labyrinth barrier, inducing a cytotoxic effect on the cochlea (15). Further damage to the inner ear may occur from occlusion of the blood vessels supplying the inner ear. The vascular occlusion may result from septic emboli or thrombus formation, with resultant cochlear hypoxia and ischemia and neural damage (3, 7).

Meningitis-associated hearing loss affects the development of communication skills, particularly in cases of pre-lingual hearing loss, and can lead to the speech and language regression of the child to an earlier stage of development (16). The goal of this study was to determine the hearing thresholds across all speech frequencies in post-bacterial meningitis and the risk factors associated with developing hearing loss in African children with bacterial meningitis.

## Methods

This is a prospective longitudinal study of all the children managed for bacterial meningitis in the University College Hospital Ibadan, Nigeria, between January 2016 and June 2017. Institutional ethical clearance was obtained from the joint University of Ibadan and University College Hospital, Ibadan Ethics Committee (UI/EC/15/0113), and children that met the inclusion criteria were recruited consecutively, after obtaining consent from the caregivers. Children aged between 2 months and 14 years were recruited into the study after confirmation of a diagnosis of bacterial meningitis by a pediatric neurologist as defined by the World Health Organization (WHO) guidelines based on symptoms, signs, and laboratory findings (17).

Children with a prior history of hearing loss, a family history of hearing loss, or a history of treatment with ototoxic medication previously or as part of current treatment regimen and those with a chronic medical condition were excluded from the study. The controls were healthy children who were matched for sex and age with bacterial meningitis children. They were recruited from children of members of staff and other children who accompanied their parents to the hospital complex.

A *pro forma* was used to obtained data from the mothers/caregivers on socioeconomic status, the presenting complaints, duration of symptoms, history of hearing loss or ear infection, immunization history, and drug history, with emphasis on the medication taken prior to presentation for the index illness.

For laboratory results, cerebrospinal fluid analysis, full blood count, and blood chemistry were also documented. All diagnosis of bacterial meningitis was made in agreement with the WHO guideline (17). All the patients were managed according to the standard hospital protocol for the management of childhood bacterial meningitis with intravenous third-generation cephalosporin (18).

Auditory assessment was done for all the survivors within the 48 h prior to hospital discharge, in a well-lit ventilated soundproof room, after an initial otoscopy. Tympanometry was done for children with suspected middle ear pathology following the otoscopy. This was also repeated at the follow-up clinic at 1 month after hospital discharge, irrespective of the initial hearing assessment results. An automated screening with otoacoustic emission (OAE) test was done using a Labat hand-held OAE machine (Echolab, Mogliano Veneto, Italy), followed by a diagnostic auditory brain stem response (ABR) test using an MB II classic device (MAICO, Berlin, Germany). This auditory evaluation was also done for the controls. Each ear was assessed separately, based on the recommendation of the American Academy of Audiology (15). The estimated hearing threshold was classified according as: mild hearing loss [wave V was not absent until the 30- to 40-dB hearing level (HL)]; moderate hearing loss (wave V was not absent until the 50- to 60-dB HL); severe hearing loss (wave V was not absent until the 70- to 90-dB HL); and profound hearing loss (wave V was not absent until a >90-dB HL) (11). The predictors of hearing loss were determined by multiple logistic regression. All the variables were analyzed separately initially, and those variables that were significantly associated with hearing loss were put into the regression model.

## Results

**Figure 1:**
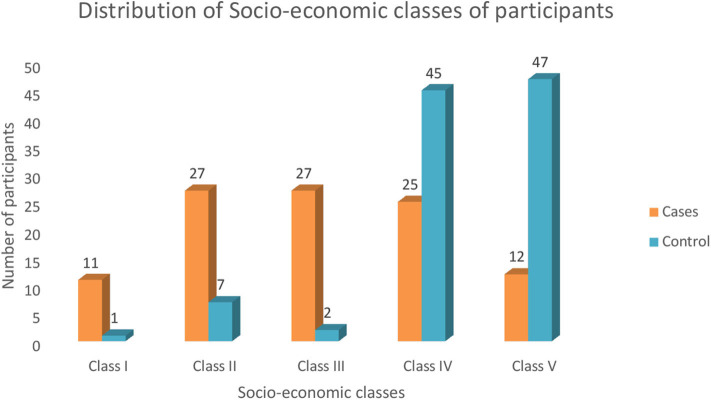
Socioeconomic classes of the participants.

There were 102 cases of bacterial meningitis: 57 males and 45 females, with a male-to-female ratio of 1.2:1. The control group was made up of 55 males and 47 females. The age of both the cases and the controls ranged from 2 months to 14 years, with a median age of 4 years among the cases, while among the control subjects the median age was 5.9 years. The age distribution of the study subjects and the socioeconomic class distribution are shown in Table 1 and Figure 1, respectively.

**Table 1:** Age distribution of the participants.

**Age range**	**Count (%)**	
	**Cases**	**Controls**
≤5 years	58 (56.9)	45 (44.2)
6–10 years	28 (27.5)	38 (37.2)
≥11 years	16 (15.6)	19 (18.6)

Table 2 shows the duration of illness among the patients before hospital presentation. Evaluation of hearing among the cases showed that 183 (89.7%) ears passed the OAE test, while 21 (10.3%) ears failed (five children failed in both ears, while 11 children failed the OAE test in one ear); two ears among those who failed the OAE test had type B tympanogram. Hearing evaluation among the controls revealed that 198 (97%) ears passed the OAE test, while 6 (3%) ears failed the OAE test (two children failed in both ears, while two children failed the OAE test in one ear). There was a significant difference in the OAE thresholds among the children with bacterial meningitis and the control group (*p* = 0.04).

**Table 2:** Duration of meningitis illness of the cases prior to hospital admission.

**Duration of illness**	**Frequency of cases, *n* (%)**
<7 days	89 (87.3)
8–14 days	6 (5.9)
>14 days	7 (6.9)
Total	102 (100)

Abnormal ABR-estimated thresholds were seen in 55 ears among the cases (24 children had bilateral and seven children had unilateral abnormal ABR thresholds), giving a prevalence of 30.4%. Among the controls, 11 ears had abnormal ABR thresholds (four children had bilateral and three children with unilateral involvement), giving a prevalence of 6.9%. There was a statistically significant difference in the prevalence of hearing loss between the cases and the controls (*p* = 0.01). Table 3 shows the classification according to the severity of hearing loss, while Table 4 shows the relationship of the OAE and ABR results of each ear. All cases of hearing loss were stable at 1 month reassessment, with the exception of a single case among the post-meningitis children, who had a type B tympanogram bilaterally with improvement from bilateral mild hearing loss to normal hearing threshold and improvement in the OAE thresholds, from fail to pass at 1 month.

**Table 3:** Distribution of the auditory brain stem response (ABR) hearing thresholds among survivors post-meningitis.

**ABR hearing thresholds**	**Frequency, *n* (%)**
Normal	149 (73.0)
Mild	10 (4.9)
Moderate	9 (4.4)
Severe	17 (8.3)
Profound	19 (9.3)
Total	204 (100)

**Table 4:** A comparison of the otoacoustic emission (OAE) and auditory brain stem response (ABR) results in the study participants.

		**OAE results**			
		**Pass**		**Fail**	
		**Cases**	**Controls**	**Cases**	**Controls**
ABR	Normal	142	193	7	0
	Abnormal	41	5	14	6
Total		183 (89.7%)	198 (97%)	21 (10.3%)	6 (3%)

Multiple logistic regression showed the following predictors of hearing loss: duration of illness (*OR* = 1.554), abnormal packed cell volume (*OR* = 0.933), white blood cell count (*OR* = 1.080), and random blood sugar (RBS)/cerebrospinal fluid (CSF) sugar ratio (*OR* = 1.005; Table 5). Thus, a unit increase in any of these four variables will likely cause the occurrence of hearing loss in children post-bacterial meningitis.

**Table 5:** Predictors of hearing loss in survivors of pediatric bacterial meningitis.

**Predictors of hearing loss**	***B***	**SE**	**Wald**	***df***	**Sig**.	**Exp. (*B*)**	**95% CI for Exp. (*****B*****)**	
							**Lower**	**Upper**
Duration of illness before hospital admission	0.441	0.162	7.437	1	0.006	1.554	1.132	2.134
WBC	−0.077	0.023	11.092	1	0.001	1.080	1.032	1.131
RBS/CSF sugar	0.005	0.002	4.735	1	0.030	1.005	1.000	1.009
PCV	−0.707	0.030	5.523	1	0.019	0.933	0.880	0.989

*WBC, white blood count; RBS, random blood sugar; CSF, cerebrospinal fluid; PCV, packed cell volume*.

## Discussion

The prevalence of hearing loss post-bacterial meningitis seen in this study is 30.4%. This rate is much higher than the prevalence rates of 2.4 and 13.7% reported from the United Kingdom and the USA, respectively (13, 19); however, similar prevalence rates of 22–44% were reported from several LMICs (11, 18, 20). The wide difference in the prevalence rates could be due to better immunization practices and a subsequent reduction in susceptibility to fatal disease, better health-seeking habits, and early hospital presentation in the USA and the UK. It is also possible that the virulence of the bacterial agents and resistance to antibiotics by some of the etiological agents in LMICs may be responsible for the difference in the prevalence rates (18).

There were more cases of bilateral hearing loss, probably due to the simultaneous involvement of both ears during the infection (21, 22). The bilateral involvement may increase the likelihood of the development of disabling hearing loss.

A third of the ears that failed the OAE test in the study population had normal ABR hearing thresholds, suggesting possession of normal hearing thresholds despite a probable cochlea pathology. This observation had been attributed to lesions occurring in both the cochlea and the auditory nerve during bacterial meningitis (11). Richardson et al. (13) proposed the auditory site of lesion in bacterial meningitis to be solely the cochlea, probably because, in that study, all the cases with permanent sensorineural hearing loss had abnormal ABR and OAE thresholds. However, in this study, 33.3% of the ears had normal ABR hearing thresholds, but failed the OAE test, suggesting dual involvement of the cochlea and the auditory nerve as possible lesion sites. The reason for this observation is not immediately obvious, though the difference in the health-seeking habits of the study population and possibly the virulence of the etiological agents may play a role.

Auditory neuropathy spectrum disorder (ANSD) is diagnosed in the presence of a normal OAE response with an abnormal ABR threshold (23, 24), which is suggestive of either a pathology involving the inner hair cells, abnormal synapsis between the inner hair cells and the spiral ganglion neurons, or a neuropathy along the auditory pathway (25). Although ANSD can occur in the absence of a medical illness, infectious diseases (such as bacterial meningitis) are a known risk factor for its development (26). In this study, 41 ears had abnormal ABR thresholds with normal OAE responses, suggesting an ANSD prevalence of 20%.

Fluctuations in the sensorineural hearing loss following bacterial meningitis in children have been reported in the literature (13, 16). Some studies have shown a fluctuation in hearing loss of up to 22% (19, 22). We observed a single case of change in hearing threshold, and this was a conductive hearing loss (likely due to otitis media with effusion). All other cases of hearing loss were stable. Studies on survivors of bacterial meningitis had used various follow-up periods varying from a few weeks to 4 years following discharge from the hospital (19, 20, 22). It is possible that the relatively short follow-up period in this study (4 weeks) may account for the low rate of fluctuation in hearing impairment.

The identified predictors of hearing impairment in this study were anemia, leukocytosis, hypoglycorrhachia, and random blood sugar. Previous studies ascertained multiple factors as predictors of hearing impairment. These factors include the following: (1) illness >24 h before intervention (13); (2) severe illness warranting ICU admission (11); (3) raised intracranial pressure at admission; (4) male gender; (5) reduced CSF glucose; (6) *S. pneumoniae* as the infective organism; (7) nuchal rigidity (21); (8) seizure and fever; (9) low Glasgow Coma Scale value (22); (10) age <2 years; (11) elevated CSF protein; and (12) abnormal sodium level (22). The wide variations in the reported predictors could be due to differences in the study population, especially the health-seeking behavior.

The main limitation of this study is the lack of premorbid hearing thresholds of the children studied. Hearing impairment is a frequent complication of bacterial meningitis, and the probable site of lesion is the cochlea and/or the auditory nerve. The factors that worsen hearing loss in children with bacterial meningitis are anemia, leukocytosis, hypoglycorrhachia, and duration of illness before appropriate medical intervention.

## Data Availability Statement

All datasets generated for this study are included in the article/supplementary material.

## Ethics Statement

The studies involving human participants were reviewed and approved by University of Ibadan, University College Hospital Ibadan Ethics Committee. Written informed consent to participate in this study was provided by the participants' legal guardian/next of kin.

## Author Contributions

MJ, AA, IL, and ON contributed to conception and designed the study. MJ, AA, and SO drafted the manuscript. All the authors contributed to manuscript revision and approved the manuscript.

## Conflict of Interest

The authors declare that the research was conducted in the absence of any commercial or financial relationships that could be construed as a potential conflict of interest.
